# Nurses' Ethical Competence, Perceived Ethical Climate and Stereotypes of Older Adults: A Cross Sectional Survey

**DOI:** 10.1111/opn.70093

**Published:** 2026-07-11

**Authors:** Anna‐Liisa Arjama, Mari Kangasniemi, Riitta Suhonen

**Affiliations:** ^1^ Department of Nursing Science University of Turku Turku Finland; ^2^ Turku University Hospital Wellbeing Services County of Southwest Finland Turku Finland

**Keywords:** ethical climate, ethical competence, long‐term care, older adult, quantitative, stereotypes, survey

## Abstract

**Introduction:**

Nurses' ethical competence and the ethical climate of healthcare settings are critical in promoting high‐quality and individualised care. However, these factors may also contribute to various stereotypes that healthcare professionals hold towards older adults, potentially hindering the recognition of patients' individuality. The aim of this study was to investigate possible associations between nurses' characteristics, ethical competence, perceived ethical climate and stereotypes towards older adults in long‐term care settings.

**Methods:**

This was a cross sectional survey study. Nurses working in long‐term care settings for older adults in Finland participated in the study between 2024 and 2025. The survey included three self‐administered instruments: the Ethical Competence Questionnaire, [Hospital] Ethical Climate Survey and Stereotype Content and Strength Survey. The data were analysed using multiple regression models to identify associations between nurses' characteristics, ethical competence, perceived ethical climate and stereotypes regarding older adults.

**Results:**

A total of 409 nurses participated. The participants rated their ethical competence and perceived ethical climate of their workplace as moderate or good. Both factors were statistically significantly associated with positive stereotypes towards older adults but not with negative stereotypes. Most participants (89%) held employee positions and had vocational degrees (65%). Younger age was associated with stronger stereotypes. When the independent variables of ethical competence and ethical climate were tested together, ethical competence alone no longer explained positive stereotypes, but ethical climate did, even after adjusting for age.

**Conclusion:**

This study confirms that nurses' ethical competence and perceived ethical climate support each other. Strengthening these elements can help alleviate stereotypes about the residents in long‐term care settings. Maintaining and developing these factors can promote the delivery of high‐quality, individualised care. To mitigate stereotypes regarding LTCS residents, the nurse manager can create structures that strengthen ethical competence and support an ethical climate. Further research is needed to clarify the determinants that inform nurses' assessments of stereotypes towards older adults in long‐term care settings.

**Implications for Practice:**

Ethical climate is associated with the content and strength of stereotypes that nurses have towards older adults. Therefore, maintaining and developing an ethical climate is important. Every nurse can promote an ethical climate by improving their ethical competence and promoting positive relationships with different stakeholders. In addition, it is worth creating structures in organizations that strengthen nurses' ethical competence and ethical climate.

## Summary

**What does this research add to existing knowledge in gerontology?:**

- Nurses rated their ethical competence and perceived ethical climate as moderate or good in long‐term settings for older adults.
- In long‐term care settings, nurses' stereotypes towards older adults are relatively neutral.
- Nurses' ethical competence, ethical climate and stereotypes towards older adults are statistically significantly associated.
- Nurses' perceived ethical climate is associated with the appearance of stereotypes regardless of the nurses' age, experience or other individual characteristics.

**What are the implications of this new knowledge for nursing care for and with older adults?:**

- Every nurse working in long‐term care settings for older adults can contribute to the ethical climate by maintaining their ethical competence and fostering positive relationships with various stakeholders.
- Nurses' high levels of ethical competence and ethical climate provide a solid foundation for implementing high‐quality individualised care.

**How could the findings be used to influence practice, education, research, and policy?:**

- By creating structures that strengthen nurses' ethical competence and the ethical climate, it is possible to improve the quality of individualised care.
- Ethical competence is essential for nurses in the long‐term care of older adults. It needs to be strengthened during nursing education and continuously maintained throughout working life.
- Research on stereotypes about older adults more broadly in society could be valuable in aging societies.

## 1 Introduction

In long‐term care settings (LTCSs) for older adults, nurses face ethical issues related to resident self‐determination, collaboration with peers, organisation and leadership, and societal issues (Podgorica et al. 2021; Suhonen and Stolt 2024). In Finland, nursing care in LTCS is intended for people who need constant care and attention (Social Welfare Act 2022). The service is provided by trained healthcare professionals and addresses the needs of growing number of people who are no longer able to live independently at home. Unlike hospitals, residents are offered home‐like care that comprehensively takes into account their individual preferences (Ministry of Social Affairs and Health 2012). The family members can participate in daily care, the care relationship between nurses and residents may be long‐lasting and close, and often continues through the resident's end‐of life phase (Choe et al. 2018; Lopez et al. 2013; Teeri et al. 2006). Ethical issues in long‐term care are also raised by internal and external challenges within nursing homes, such as staff friction and the availability of skilled professionals, as well as global challenges such as the COVID‐19 pandemic (Benvenuti et al. 2021; Choe et al. 2018; Nikunlaakso et al. 2022). Thus, they need ethical competence and a supportive ethical climate to be able to face these challenges and provide high‐quality and individualised care (Koskenvuori et al. 2019; Poikkeus et al. 2020; Suhonen et al. 2015). However, older adults worldwide have experienced that healthcare professionals can hold stereotypes towards them and do not necessarily recognise their individuality (Ayalon 2018; Crutzen et al. 2022; Fernández‐Puerta et al. 2024; WHO 2025).

*Ethical competence* in nursing practice refers to a nurse's familiarity with the legislation, values and principles related to their work. It encompasses their ability to reflect on ethical issues, take ethical perspectives into account in decision‐making and act ethically. Maintaining ethical competence requires continuous ethics education so that the nursing professional can meet the challenges of nursing, ensure patient safety and high‐quality care, and maintain their own professional well‐being (Grady et al. 2008; Jakobsen et al. 2023; Lan et al. 2017; Poikkeus et al. 2020). *Ethical climate* denotes a shared understanding of what is ethically correct and how ethical decisions are made. It includes the circumstances in which employees can engage in ethical reflection, and how ethical issues are addressed in the organisation (Okumoto et al. 2022; Olson 1995; Suhonen et al. 2014). A stereotype is a widely held and oversimplified and fixed belief or image about a particular group of people. *Stereotypes regarding older adults* are generalised beliefs that portray older adults as a homogenous group. Stereotypes may underestimate or overestimate the characteristics of an individual and cause stigmatisation based on appearance (Bodner 2009; Carlson et al. 2020; Dobbs et al. 2008; Voss et al. 2017). Stereotypes may be harmful especially when they are highly negative, because they can operate unconsciously, so among nursing professionals they may undermine individual encounters with older adults and equal care (Crutzen et al. 2022; Levy 2009).

In previous studies, nurses have assessed their ethical competence as average or good and have considered ethical behaviour and action to be the strongest area of their ethical competence. Individual and organisational support have a significant impact on nurses' ethical competence (Poikkeus et al. 2020). Further, nurses have assessed their ethical climate as being moderate or good, with relationships with peers being the strongest area of it. Relationships with doctors have been rated lowest, but still on a moderate or good level (Suhonen et al. 2014). Ethical climate has been identified as a prerequisite for boosting the ethical competence of nurses (Yu et al. 2025) which makes this factor significant in strengthening the effectiveness of care (Tang et al. 2024). The associations between ethical competence and ethical climate have been shown to be reciprocal (Yu et al. 2025) and there are indications that improving ethical competence and the ethical climate can mitigate stereotypes regarding older adults (Fernández‐Puerta et al. 2024; Suhonen et al. 2014). The research results on the content and strength of nurses' stereotypes regarding older adults are conflicting but negative perceptions have been found to increase when nurses have little knowledge and experience of interacting with the older adults (Allué‐Sierra et al. 2023; Crutzen et al. 2022). Negative stereotypes have been found to link with potential ageism leaving older adults with reduced autonomy or reduced inclusion (Carlson et al. 2020). Overall, nurses in LTCSs hold fewer stereotypes than healthcare students regarding older adults (Carlson et al. 2022) but more negative stereotypes than general population (Crutzen et al. 2022).

The two‐way relationships between ethical competence and ethical climate have been demonstrated in previous research (Poikkeus et al. 2018; Suhonen et al. 2014; Yu et al. 2025). Also, continuous ethics education for nurses improves ethical climate (Okumoto et al. 2022; Palsgaard et al. 2022) and reduces the potential, extremely negative stereotypes of healthcare professionals regarding older adults (Crutzen et al. 2022). Although ethical competence, perceived ethical climate and stereotypes of older adults are all associated with provision of individualised and high‐quality care (Chang et al. 2020; Poikkeus et al. 2018; Suhonen et al. 2014), this combination of factors has not been studied in the context of LTCSs.

New knowledge about ethical competence and perceived ethical climate and their connection to the content and strength of stereotypes is needed to more deeply understand the implementation of ethical nursing care. A better understanding of the associations between these concepts enables nurse managers to guide interventions in a direction that would best support individualised and high‐quality care and the well‐being of nurses. The aim of this study was to investigate the possible associations between nurses' characteristics, ethical competence, perceived ethical climate and stereotypes regarding older adults. We hypothesise that ethical competence and ethical climate are associated (Hypothesis I) and that higher ethical competence and perceived ethical climate are associated with fewer stereotypes regarding older adults (Hypothesis II).

## 2 Materials and Methods

### 2.1 Study Design

This was a cross sectional, correlational study targeting nurses working in 24‐h LTCSs for older adults in Finland during 2024–2025. The reporting of the study follows the STROBE guidelines.

### 2.2 Instruments

The survey included three validated instruments (Table 1). The Ethical Competence Questionnaire (ECQ) (27 items on a five‐step Likert‐scale) is a self‐assessing tool for nurses to rate their own competence according to how well the items describe them. A higher score indicates higher ethical competence (Poikkeus et al. 2020). The [Hospital] Ethical Climate Survey (HECS) (26 items, five‐step Likert‐scale) is used to assess nurses' relationship practices, including behaviours regarding peers, residents, managers, the organisation and physicians. Higher scores indicate that nurses perceive their work climate more ethical (Olson 1995; Suhonen et al. 2015). In the Stereotype Content and Strength Survey (SCSS) (117 items, five‐step Likert‐scale) participants were asked how many of older adults they thought could be described by the given one‐ or two‐word terms. The higher the score, the more older adults the nurses thought the stereotype applied to. Factor analysis computed for the 117 characteristics of the original instrument yielded a three‐factor model with Cronbach's alpha analyses revealing reliable scales for negative (*α* = 0.92), positive (*α* = 0.88) and physical (*α* = 0.81) stereotypes (Carlson et al. 2020).

**TABLE 1:** Description of the instruments.

	Ethical Competence Questionnaire (ECQ)	[Hopital] Ethical Climate Survey (HECS)	Stereotype Content and Strength Survey (SCSS)
Developer	Poikkeus et al. (2018) (Finland)	Olson (1995) (USA)	Carlson et al. (2020) (USA)
Validation	Poikkeus et al. (2018) (Finland)	Suhonen et al. (2015) (Finland)	Carlson et al. (2020) (USA)
Language	Finnish	English, translated to Finnish by Suhonen et al. (2015)	English, translated to Finnish for this study
Number of items	*n* = 27	*n* = 26	*n* = 117
Sub‐scales (number of items)	Knowledge of laws and regulations (*n* = 7) Knowledge of values/principles (*n* = 6) Ethical reflection (*n* = 5) Ethical decision‐making (*n* = 5) Ethical behaviour and action (*n* = 4)	Relationships with: Peers (*n* = 4) Patients (*n* = 4) Managers (*n* = 6) Organisation (*n* = 6) Physicians (*n* = 6)	Positive (*n* = 42) Negative (*n* = 49) Physical (*n* = 26)
Likert scale	1 = strongly disagree 5 = strongly agree	1 = almost never true 5 = almost always true	1 = none 5 = all
Examples of items	I am familiar with the content of the legislation regarding patient access to care.	Nurses and physicians here respect each other's opinions, even when they disagree about what is best for patients.	Cooperative Cruel Patient Crabby

The ECQ and HECS scales have been used before in the context of LTCSs and Finnish versions, which were used in this study, have been validated (Suhonen et al. 2015, Poikkeus et al. 2018). The SCSS instrument was translated into Finnish for this study (Sousa and Rojjanasrirat 2011). The items were first translated into Finnish by the author (A‐LA) and an educated linguist. After that, the items were blind back‐translated by another linguist. The translated versions were then discussed and compared to the originals by a group of eight healthcare professionals. Based on their suggestions, the final expressions were selected by the first author (A‐LA) with the help of a linguist. The final expressions were confirmed by all authors. Seven of the eight healthcare professionals involved in the translation process tested the SCSS to gain insight into its usability and response time. They found the survey a bit tedious due to the large number of items but found answering the survey rather simple as the items contained only one or two words.

### 2.3 Sample and Setting

In Finland, 21 well‐being services counties are responsible for providing long‐term care in a nursing home to older adults whose functional capacity requires continuous professional care. The counties themselves provide about half of the long‐term care services and the rest as outsourced services from private service providers. The largest professional group working in LTCS for older adults is licensed practical nurses (70%) with vocational, 180 ECTS in Social and Health Care. Other professional groups are care assistants (10%), registered nurses (7%) and supervisors (5%). Approximately 8% of the employees are auxiliars, social workers, physiotherapists and occupational therapists (Finnish Institute for Health and Welfare/monitoring report 2023 2024). In Finland, ethics is a cross‐cutting theme in nursing education, supporting professional decision‐making and the implementation of quality care.

### 2.4 Participant Recruitment and Data Collection

Two well‐being services counties were selected for this study using purposive sampling. Since the number of responses was low during the first month, an additional county was selected. The three largest private service providers in the area of these counties were also contacted for participation, and two of them were willing. A total of approximately 7500 nurses from public and private providers worked in the survey area. The researcher (A‐LA) contacted the nurse managers of the participating organisations. They were asked to act as a contact person for the research permission process, which varied by organisation, as well as for the data collection. As stated in the research permit application, the survey was required to be answered during working hours. Once research permission was received, an email containing an information sheet, a privacy note and a research link to the survey was sent to the contact person, who then forwarded it to the employees. Based on the number of employees reported by nurse managers in LTCS, the survey eventually reached approximately 4000 nurses. Data were collected between 1 August 2024 and 31 January 2025 using an electronic survey and REDCap electronic data capture tools hosted at University of Turku (Harris et al. 2009). Three reminders were sent (Phillips et al. 2016).

### 2.5 Data Analysis

The data were analysed using SPSS software (IBM SPSS Statistics 2025). Descriptive statistics were calculated for socio‐demographic and work‐related variables and the main variables. Regarding the question of education level, three groups were formed. The first group included all participants without a social or healthcare qualification. The second group consisted of licensed practical nurses with vocational training, and the third group consisted of those with a higher level of professional qualification, such as registered nurses and nurses with an academic degree. ‘Further skills education in ethics’, nurses who had answered ‘none’ or ‘little’ were grouped together as were those who answered that they had ‘quite much’ or ‘very much’ skills education in ethics.

The means and standard deviations (SDs) for the HECS and ECQ instruments were calculated using overall scale as well as on a sub‐scale level according to their theoretically suggested forms. Their internal consistency was calculated using Cronbach's alpha (*α*). To assess stereotype content (existence of a stereotype to describe a group) and strength (intensity of a stereotype regarding a specific group), response categories were grouped into 1 = ‘some, most or all’ and 0 = ‘none or few’. If the response percentage corresponding to the percentage of participants who responded ‘some or more’, was higher than 80%, it was classified as a ‘strong’ stereotype. Items with a response percentage above 66.67% were considered ‘moderate’ stereotypes. ‘Weak’ stereotypes included descriptive terms with a response percentage between 50% and 66.66% (Carlson et al. 2020). All items were used from the SCSS scale in the analysis following the three‐factor model by Carlson et al. (2020, 2022). The item ‘unemotional’ was categorised as positive in Carlson's study but the expert group in our study considered it a negative item; therefore, it was included as a negative stereotype in the analysis.

Participants who had expressed strong, moderate or weak stereotypes regarding older adults were identified. Their characteristics and self‐rated ethical competence and ethical climate were compared. The correlation between stereotypes and ethical competence and perceived ethical climate was calculated using Pearson's correlation coefficient (*r*). Associations between ethical competence and climate with stereotypes were first analysed using unadjusted linear regression and second using adjusted linear regression for age, position and service sector. Two‐sample *t*‐tests for two‐category variables, a one‐way ANOVA for multi‐category variables, and the Pearson correlation coefficient for age and experience were used to determine whether the characteristics of the nurses affected their self‐rated ethical competence, ethical climate or stereotypes regarding older adults.

### 2.6 Ethical Considerations

According to Finnish legislation, a study of this type with legally competent adults does not require ethical approval (Finnish National Board on Research Integrity TENK guidelines 2019; University of Turku, n.d.). Instead, research permits were obtained from each participating organisation, following their approval processes. Each participant provided their informed consent to participate at the first page of the survey, and this was a prerequisite for starting the survey. The information sheet stated that the survey would be answered anonymously, that background information would be collected but would not reveal the respondent's identity, that responding was voluntary and that it could be discontinued at any time. Responses given by the time of discontinuation could not be deleted later. The information collected would only be used for this study and would be stored on a secure server to which only members of the research team would have access. All participants used the same link for responding, and the responses were registered anonymously (ALLEA—All European Academies 2023).

## 3 Results

### 3.1 Participant Characteristics

Altogether, 409 nurses participated in the study (Table 2). The response rate was approximately 10%. The mean age was 47 years (range 18–68), and their work experience ranged from less than a year to 45 years. Up to 89% of the participants were employees, and 11% were supervisors. The majority (65%) had a vocational education, and more than one fourth (29%) had a degree higher than vocational level. Approximately 15% had participated in further education in ethics quite much or very much. Most of the participants (93%) worked in the public sector.

**TABLE 2:** Characteristics of participants.

	Min.	Max.	Mean	SD
Age (years) (*n* = 409)	18	68	47	11.9
Experience (years) (*n* = 409)	< 1	45	15	10.4
	***n***		**%**	
Position (*n* = 392)				
Employee	348		89	
Supervisor	44		11	
Highest degree (*n* = 400)				
No social or healthcare qualification	24		6	
Licensed practical nurse	259		65	
Registered nurse or academic degree	117		29	
Further skills education in ethics (*n* = 401)				
None or quite little	342		85	
Quite much or very much	59		15	
Service sector (*n* = 406)				
Private	28		7	
Public	378		93	

### 3.2 Level of Ethical Competence, Ethical Climate and Stereotypes Regarding Older Adults

The nurses' self‐assessed ethical competence (ECQ total) was at a good level (3.92). On a sub‐scale level, the highest mean scores were for ethical behaviour and action (mean 4.27, SD 0.57) and the lowest for knowledge of values and principles (mean 3.61, SD 0.64). Overall, the self‐assessed ethical climate (HECS total) was at a good level (4.03). On a sum variable level, the highest means were obtained for the items regarding relationships with peers (mean 4.04, SD 0.61) and the lowest for the items evaluating nurses' relationships with their organisation (mean 3.52, SD 0.71). The internal consistency of all subscales of the instruments using Cronbach's alpha (*α*) was above 0.80. Values above *α* = 0.70 are considered to be evidence of acceptable internal consistency (Table 3).

**TABLE 3:** Nurses' assessment of their ethical competence and ethical climate: means, SDs and Cronbach's alpha (*α*) on a total and sub‐scale level.

Instrument and subscales	items (*n*)	Scale	*N*	Mean	SD	*α*
**Ethical competence**	27	1–5	409	3.92	0.49	0.95
Knowledge of laws and regulations	7	1–5	408	3.89	0.59	0.90
Knowledge of values and principles	6	1–5	408	3.61	0.64	0.81
Ethical reflection	5	1–5	406	4.07	0.60	0.93
Ethical decision‐making	5	1–5	406	3.88	0.60	0.90
Ethical behaviour and action	4	1–5	407	4.28	0.57	0.89
**Ethical climate**	26	1–5	389	3.71	0.61	0.95
*Relationships of nurses with*:						
Peers	4	1–5	389	4.03	0.61	0.82
Patients	4	1–5	388	3.76	0.53	0.95
Managers	6	1–5	389	3.73	0.95	0.86
Organisation	6	1–5	388	3.53	0.71	0.86
Physicians	6	1–5	386	3.67	0.67	0.82

Over 80% of the participants expressed that they perceived some, most or all older adults as friendly, cooperative, pleasant, honest, forgetful and having grey hair. According to the SCSS, these are considered strong stereotypes. Overall, positive stereotypes met stereotype criteria more often than negative ones. According to the original instrument's three‐dimensional division, there were a total of 42 items expressing positive stereotypes, and in this study, 57% of them met the criteria of a stereotype, while the corresponding proportion of negative stereotypes (*n* = 47) was 8% (Table 4).

**TABLE 4:** Nurses' strong, moderate and weak stereotypes using SCSS instrument.

Strong stereotypes: some, most, all ≥ 80%		
Positive	Friendly	92.6
	Cooperative	90.2
	Pleasant	88.5
	Honest	80.8
Negative	Forgetful	93.9
Physical	Grey Hair	91.2
Moderate stereotypes: some, most, all = 79%–67%		
Positive	Loving	77.4
	Patient	76.2
	Humorous	75.1
	Family oriented	74.3
	Polite	73.4
	Trustful	72.6
	Agreeable	72.3
	Considerate	72.1
	Interesting	71.5
	Sociable	69.1
	Respectful	66.7
Negative	Dependent on others	74.1
	Emotional	74.0
Physical	Slow moving	78.3
	Sick	78.3
	Healthy weight	76.6
	Trouble hearing	76.1
	Trouble seeing	74.2
	Severely impaired	72.4
Weak stereotypes: some, most, all = 66%–50%		
Positive	Talkative	65.1
	Playful	63.1
	Happy	62.3
	Helpful	60.5
	Enjoys life	60.1
	Flexible	58.8
	Wise	58.5
	Intelligent	52.7
	Kind	51.4
Negative	Lonely	50.6
Physical	Sexually inactive	64.6
	Pretty/Handsome	62.5
	Unhealthy weight	52.2
	Slow thinking	51.6
	Well dressed	51.4

The ethical competence and perceived ethical climate were statistically significantly associated on the total scale level (ECQ and HECS total *r* = 0.45, *p* ≤ 0.001) and on every sub‐scale level, meaning that nurses who self‐rated their ethical competence as good also experienced the ethical climate in their workplace to be good (Table 5). The strongest association was between the sub‐scale of nurses' ethical behaviour and action and their relationships with patients (*r* = 0.42, *p* ≤ 0.001) and peers (*r* = 0.41, *p* ≤ 0.001).

**TABLE 5:** Association between ethical competence and perceived ethical climate sub‐scales.

Ethical competence	Knowledge of laws and regulations		Knowledge of values and principles		Ethical reflection		Ethical decision‐making		Ethical behaviour and action	
Ethical climate	*r*	*p*	*r*	*p*	*r*	*p*	*r*	*p*	*r*	*p*
Peers	0.28**	< 0.001	0.30**	< 0.001	0.30**	< 0.001	0.33**	< 0.001	0.41**	< 0.001
Patients	0.34**	< 0.001	0.35**	< 0.001	0.35**	< 0.001	0.39**	< 0.001	0.42**	< 0.001
Managers	0.25**	< 0.001	0.35**	< 0.001	0.22**	< 0.001	0.30**	< 0.001	0.25**	< 0.001
Organisation	0.31**	< 0.001	0.38**	< 0.001	0.23**	< 0.001	0.34**	< 0.001	0.35**	< 0.001
Physicians	0.27**	< 0.001	0.37**	< 0.001	0.25**	< 0.001	0.31**	< 0.001	0.32**	< 0.001

Abbreviations: *p*, *p*‐value; *r*, Pearson's correlation coefficient.

** Correlation is significant at the 0.01 level.

### 3.3 Associations Between Nurses' Characteristics, Their Ethical Competence, Ethical Climate and Stereotypes Regarding Older Adults

The age of the participants was statistically significantly associated with ethical competence (*p* = 0.003) that the younger the nurses' age, the better the self‐rated ethical competence. Also, the younger the age, the more both positive (*p* = 0.002) and negative (*p* = 0.001) stereotypes were present (Table 6). The more work experience participants had, the fewer negative stereotypes they had (*r* = −0.17, *p* = 0.002). Participants' educational background was associated with ethical competence. Nurses with higher degrees had better ethical competence (mean difference 0.36, *p* = 0.002). Participants working in the private sector rated their perceived ethical climate (mean difference 0.42, *p* = 0.008), ethical competence (mean difference 0.17, *p* ≤ 0.001) and positive stereotypes (mean difference 0.25, *p* = 0.02) with higher values than participants in the public sector. Position within the organisation was associated with ethical climate (mean difference 0.30, *p* = 0.003), ethical competence (mean difference 0.33, *p* ≤ 0.001) and positive stereotypes regarding older adults (mean difference 0.25, *p* = 0.004). The supervisors rated their ethical competence and ethical climate more positively than the care workers did; likewise, the supervisors held more favourable stereotypes about older adults. Altogether 57 participants (15%) reported having participated in a considerable amount (‘quite much’ or ‘very much’) of ethics' education during their working years. The participants rated their ethical competence (mean difference 0.34, *p* < 0.001) and ethical climate (mean difference 0.21, *p* = 0.01) significantly higher than those who reported little or no ethics' education during their working years.

**TABLE 6:** Associations between nurses' characteristics with ethical competence, perceived ethical climate and positive and negative stereotypes regarding older adults.

	ECQ			HECS			Positive stereotypes			Negative stereotypes		
	*n*	*r*	*p*	*n*	*r*	*p*	*n*	*r*	*p*	*n*	*r*	*p*
Age	409	−0.156	**0.003**	389	−0.07	0.19	368	−0.17	**0.002**	368	−0.175	**0.001**
Experience	409	−0.083	0.10	399	−0.047	0.36	368	−0.03	0.54	368	−0.17	**0.002**
	***n***	**Mean (SD)**	***p***	***n*** ^c^	**Mean (SD)**	***p***	***n***	**Mean (SD)**	***p***	***n***	**Mean (SD)**	***p***
Highest degree												
Lower than vocational	24	3.64^a^, ^b^ (0.52)	**0.002**	22	3.50 (0.72)	0.19	21	2.85 (0.66)	0.14	21	2.44 (0.51)	0.15
Vocational	259	3.90^a^ (0.48)		250	3.69 (0.60)		234	2.68 (0.51)		234	2.29 (0.36)	
Higher than vocational	117	4.00^b^ (0.47)		108	3.76 (0.59)		105	2.77 (0.50)		105	2.28 (0.27)	
Service sector												
Public	378	3.90 (0.49)	**0.008**	361	3.68 (0.60)	**< 0.001**	340	2.70 (0.52)	**0.02**	340	2.29 (0.33)	0.28
Private	28	4.07 (0.53)		25	4.10 (0.44)		25	2.95 (0.55)		25	2.37 (0.47)	
Position												
Employee	348	3.88 (0.47)	**< 0.001**	335	3.66 (0.61)	**0.003**	317	2.69 (0.52)	**0.004**	317	2.31 (0.36)	0.093
Supervisor	44	4.21 (0.40)		40	3.96 (0.50)		39	2.94 (0.42)		39	2.21 (0.24)	
Ethics' education												
None/little	342	3.86 (0.49)	**< 0.001**	324	3.67 (0.60)	**0.01**	309	2.69 (0.50)	0.11	309	2.30 (0.34)	0.88
Quite/very much	59	4.20 (0.42)		57	3.88 (0.63)		52	2.81 (0.58)		52	2.29 (0.38)	

Abbreviation: *p*, *p*‐value.

^a^ *p* = 0.026 between ‘lower than vocational’ and ‘vocational’, Tukey's test.

^b^ *p* = 0.002 between ‘lower than vocational’ and ‘higher than vocational’, Tukey's test.

^c^ *Note:* Values are in bold to emphasize that they are statistically significant (< 0.05).

Linear regression showed that nurses' ethical competence (*β* = 0.23, *p* ≤ 0.001) and perceived ethical climate (*β* = 0.27, *p* ≤ 0.001) were statistically significantly associated with positive stereotypes of older adults but not with negative stereotypes (Table 7). When the independent variables of the ECQ and HECS were tested together, nurses' ethical competence alone no longer explained the positive stereotypes (*p* = 0.111), but perceived ethical climate did (*p* ≤ 0.001).

**TABLE 7:** Associations between ethical competence, perceived ethical climate and stereotypes of older adults measured with the ECQ, HECS and SCSS and adjusted with background variables.

Explanatory factor	Positive stereotypes				Negative stereotypes			
	Unadjusted		Adjusted^a^		Unadjusted		Adjusted^b^	
	*β* (CI)	*p*	*β* (CI)	*p*	*β* (CI)	*p*	*β* (CI)	*p*
ECQ	0.23 (0.12–0.34)	< 0.001	0.10 (−0.02–0.22)	0.11	0.01 (−0.08–0.09)	0.89	0.03 (−0.06– −0.12)	0.50
HECS	0.27 (0.19–0.36)	< 0.001	0.24 (0.14–0.33)	< 0.001	−0.04 (−0.11–0.03)	0.23	0.067 (−0.13–0.00)	0.051

Abbreviations: *β*, regression coefficient; CI, confidence interval; *p*, *p*‐value.

^a^ Adjusted for age, position, employer.

^b^ Adjusted for age.

On the sub‐scale level of ethical competence, the strongest association, albeit low, with positive stereotypes appeared for knowledge of values and principles (*r* = 0.21, *p* ≤ 0.001) and ethical decision‐making (*r* = 0.20, *p* = 0.001). Regarding the sub‐scales of ethical climate, the strongest associations with positive stereotypes were observed for relationships with managers (*r* = 0.028, *p* ≤ 0.001) and with the organisation (*r* = 0.30, *p* ≤ 0.001) (Table 8).

**TABLE 8:** Associations between nurses' ethical competence, perceived ethical climate and the negative, positive and physical stereotypes they hold.

	Negative stereotypes			Positive stereotypes			Physical stereotypes		
	*r*	*p*	*n*	*r*	*p*	*n*	*r*	*p*	*n*
Ethical competence									
Knowledge of laws and regulations	−0.01	0.75	368	0.15**	0.003	368	0.63	0.23	365
Knowledge of values and principles	−0.02	0.78	368	0.21**	< 0.001	368	−0.02	0.77	364
Ethical reflection	−0.01	0.80	367	0.16**	0.003	367	0.02	0.78	365
Ethical decision‐making	−0.06	0.27	367	0.20**	< 0.001	367	−0.02	0.77	364
Ethical behaviour and action	−0.09	0.10	368	0.13*	0.01	368	−0.01	0.90	362
Ethical climate, *Relationship with*									
Peers	−0.06	0.27	367	0.21**	< 0.001	367	−0.02	0.76	366
Patients	−0.04	0.48	366	0.27**	< 0.001	366	−0.02	0.75	366
Managers	−0.07	0.20	367	0.28**	< 0.001	367	0.10	0.05	365
Organisation	−0.03	0.57	366	0.30**	< 0.001	366	−0.04	0.51	365
Physicians	−0.06	0.25	364	0.26**	< 0.001	364	0.08	0.15	366

*Note:* Values are bolded to emphasize that they are statistically significant.**Correlation is significant at the 0.01 level.

Abbreviations: *n*, number of participants; *p*, *p*‐value; *r*, Pearson's correlation.

## 4 Discussion

According to the findings of this study, nurses in LTCSs assess their ethical competence and perceived ethical climate as good, and the stereotypes they hold towards older adults are generally neutral. These conditions facilitate the provision of high‐quality, individualised care. This study also confirms that the levels of nurses' ethical competence and perceived ethical climate are mutually supportive, confirming Hypothesis I. A novel finding of this study is that a higher level of perceived ethical climate among nurses is associated with milder stereotypes towards older adults, confirming Hypothesis II. It is noteworthy that perceived ethical climate is associated with the appearance of stereotypes regardless of the nurses' age, experience or other individual characteristics. Therefore, strengthening both ethical competence and ethical climate may contribute to reducing stereotypes about the residents in LTCSs.

The findings of this study, indicating that the self‐assessed *ethical competence* of the nurses is at on moderate or good level, supports previous literature in the context of acute care hospitals (Poikkeus et al. 2020). The area most highly rated by the nurse participants was their *ethical behaviour*, and the weakest was *knowledge of values and principles*, which was still moderate. Previous research has shown that nurses feel they are unable to care for residents in accordance with the principles that guide their work due to their workload (Arjama et al. 2023; Nikunlaakso et al. 2022). The lower self‐assessment of knowledge regarding values and principles that guide their work may reflect a lack of awareness of these values or principles and thus support a reliance on intuition and personal expertise. The dynamic and complex nature of care situations can hinder the ability to identify which values and principles are applicable at any given time.

Based on the findings of this study, nurses generally did not hold extremely positive or negative stereotypes about older adults, although stereotypes regarding older adults might be common in society (Fernández‐Puerta et al. 2024; WHO 2025). Only one strong negative stereotype ‘forgetful’ (94%) and two moderate stereotypes ‘emotional’ (74%) and ‘dependent on others’ (74%) emerged. Some moderate physical stereotypes, which can be interpreted as negative, were ‘trouble hearing and seeing’ (76%, 74%), ‘slow moving’ (78%), ‘sick’ (78%) and ‘severely impaired’ (72%). However, over 50% of the participants believed that some or most older adults are lonely, sexually inactive, or slow thinking or have an unhealthy weight, meaning that over half of the nurses viewed older adults as passive and dependent on others. This raises the question of whether nurses' stereotypes are based on their societal images of older adults in general or whether they base their views on their own experiences with residents in LTCS. It is also possible that nurses' stereotypes reflect their concern for residents nearing the end of life, particularly those suffering from loneliness and low functional capacity. Previous research has shown conflicting information about the stereotypes that healthcare professionals have about older adults (Crutzen et al. 2022; Fernández‐Puerta et al. 2024). The overall neutral stereotypes can be interpreted as the fact that the majority of LTCS workers in Finland have a vocational degree, which gives good qualifications to care work. Overall, ethics education has a significant positive influence on professionals' moral confidence, moral action and use of ethics resources (Grady et al. 2008; Poikkeus et al. 2018). Other possible interpretations may include positive, daily encounters with residents (Crutzen et al. 2022; Palsgaard et al. 2022), but further qualitative research is needed to clarify their associations.

In this study, younger age was associated with stronger stereotypes. To encourage young nursing students to pursue careers in LTCSs for older adults, it is necessary to include a curriculum in nursing education that emphasises core nursing values and the reduction of stereotypes regarding older adults (Allué‐Sierra et al. 2023; Crutzen et al. 2022; Palsgaard et al. 2022; Pang et al. 2025). However, positive perceptions towards the older adults do not necessarily indicate a willingness to work among with this population (Pang et al. 2025). Overall, interpretations of professionals' stereotypes towards the older adults are contradictory (Allué‐Sierra et al. 2023; Carlson et al. 2022; Crutzen et al. 2022; Palsgaard et al. 2022). Some studies suggest that professionals hold stereotypical views towards older adults than the general population do (Crutzen et al. 2022). This may be because they work daily with people who require care, which may influence their thinking without necessarily reflecting attitudinal bias. Further research is needed to clarify the meaning behind LTCS nurses' assessments of stereotypes towards older adults. Such clarification would not negate older adults' experiences of not being seen as individuals, but it could clarify the conflicting interpretations of stereotypical thinking among professionals in general.

The sample covered all typical occupational groups of nursing professionals working in LTCSs for older adults. According to the results, higher self‐rated ethical competence and climate were associated with more positive stereotypes of older adults. These findings highlight the importance of an ethical climate in promoting neutral perceptions towards older adults. The ethical climate of an organisation is produced by individuals' behaviours and is connected to the content and strength of stereotypes (Carlson et al. 2020; Olson 1995; Suhonen et al. 2014). Every nurse in an LTCS can contribute to the ethical climate by maintaining their own ethical competence and paying attention to relationships with different stakeholders. By creating structures that strengthen the ethical competence that supports ethical climate, nurse managers can influence the stereotypes of LTCS residents. This can include regular ethical reflection on situations related to residents' care as well as providing individual support to staff. Further research is needed on the variables that might mediate the relationship between ethical climate and stereotypes, for example, attitude towards one's own aging (Voss et al. 2017), perceived ethical distress (Gherman et al. 2023), implemented standards for behaviour within the work team (Silén et al. 2012) or organisations' support for ethical competence (Poikkeus et al. 2018).

### 4.1 Strengths and Limitations

The strengths of the study relate to the sample, target group and methods. Successful research into ethics requires well‐operationalised and thus valid instruments as ethical issues are abstract in nature and related to the respondents' views of the world. The cross sectional design pointed out the hypothesised relationships. Validated instruments were used and regarding the SCSS instrument, the original instrument's three‐dimensional division and the variables placed within these dimensions were used. The response rate for surveys is expected to be low, and therefore, we aimed for 300–500 responses based on the thumb rule of 5–10 responses per items and achieved this by expanding the survey to a third wellbeing service county (Phillips et al. 2016). Due to the low response rate, the results should be generalised with caution although in this study the participants' characteristics largely reflected the target group, as most had a vocational education. The researchers did not have access to recruit the participants; instead, the emails were delivered through a contact person from each organisation. Because of that and a 2‐week layoff affecting all employees in one of the three participating wellbeing services counties, it is not certain whether the survey reached all employees at LTCS, so the response rate is based on a rough estimate. Missing data were low (< 5%) and were excluded from the analyses due to their small number (Tables 6 and 8). However, several limitations of the study should be acknowledged. First, the participants of this study had, on average, a higher level of education than the broader study population, which may have distorted the assessment of ethical competence and climate. In addition, supervisors (11%) and public sector employees (93%) were overrepresented in the study. The finding that supervisors and nurses in the private sector rated their ethical competence and climate more positively should not be unequivocally generalised. Another limitation concerns the use of self‐assessment instruments which are associated with social desirability bias, increasing the tendency of participants to present a positive self‐image (Van der Mortel 2008). When assessing one's own behaviour or relationship with peers, participants may assess their performance within a group context, contributing to positively skewed results. Perceived ethical climate was associated with neutral stereotypes. Ethical climate was assessed based on how the nurses perceived their workplace relationships. It is possible that nurses who are more extroverted in their relationships with other people have more positive attitudes towards older adults and at the same time experience a more ethical workplace climate and have higher ethical competence. The HECS instrument has been validated in the LTCS environment, but the associations observed in this study may require further research.

## 5 Conclusion

Nurses' ethical competence and perceived ethical climate were found to be at a good level and their stereotypes towards older adults were generally moderate. These findings suggest that nurses are well equipped to provide high‐quality, individualised care in LTCSs for older adults. Perceived ethical climate is associated with the presence of stereotypes regardless of the nurses' age, experience or other individual characteristics. Therefore, to mitigate stereotypes related to LTCS, the nurse manager can create structures and enable ongoing ethical education for nursing professionals to strengthen ethical competence and support an ethical climate. Further research is needed to clarify the meaning behind LTCS nurses' assessments of stereotypes towards older adults.

## Author Contributions

Study design: A.‐L.A., M.K. and R.S. Data collection: A.‐L.A. Data analysis: A.‐L.A., M.K. and R.S. Manuscript preparation: A.‐L.A., M.K. and R.S.

## Funding

This work was funded by Finnish Nurses Association, Akavan sairaanhoitat ja Taja ry, Konung GustafV:s och Drottning Victorias Stiftelse and the Wellbeing Services County of Southwest Finland.

## Ethics Statement

This survey targeted adult professionals and no ethical board approval was needed (ALLEA—All European Academies, 2023). Rather, a research permit was obtained from each participating organisation, following each organisation's own approval processes. All participants provided their informed consent before participating. Responses were registered anonymously. Data generated during the study will be stored on a secure server.

## Conflicts of Interest

The authors declare no conflicts of interest.

## Data Availability

The data that support the findings of this study are available on request from the corresponding author. The data are not publicly available due to privacy or ethical restrictions.
